# Vascular calcification and response to neoadjuvant therapy in locally advanced rectal cancer: an exploratory study

**DOI:** 10.1007/s00432-021-03570-1

**Published:** 2021-03-12

**Authors:** Katrina A. Knight, Ioanna Drami, Donald C. McMillan, Paul G. Horgan, James H. Park, John T. Jenkins, Campbell S. D. Roxburgh

**Affiliations:** 1 Academic Unit of Colorectal Surgery, University of Glasgow, Glasgow Royal Infirmary, Level 2, New Lister Building 10, 16 Alexandra Parade, Glasgow, G31 2ER UK; 2 St Mark’s Hospital and Academic Institute, London, UK; 3 Institute of Cancer Sciences, University of Glasgow, Glasgow, UK

**Keywords:** Calcification, Physiologic, Rectal neoplasms, Neoadjuvant therapy, Chemoradiotherapy, Hypoxia

## Abstract

**Purpose:**

Patients with locally advanced rectal cancer (LARC) may experience a clinical complete response (cCR) to neoadjuvant chemoradiotherapy (NACRT) and opt for non-operative management. Pathological factors that relate to NACRT response have been well described. Host factors associated with response, however, are poorly defined. Calcification of the aortoiliac (AC) vessels supplying the rectum may influence treatment response.

**Methods:**

Patients with LARC having NACRT prior to curative surgery at Glasgow Royal Infirmary (GRI) and St Mark’s hospital (SMH) between 2008 and 2016 were identified. AC was scored on pre-treatment CT imaging. NACRT response was assessed using pathologic complete response (pCR) rates, tumour regression grades (TRGs), the NeoAdjuvant Rectal score and T-/N-downstaging. Associations were assessed using Chi-squared, Mantel–Haenszel and Fisher’s exact tests.

**Results:**

Of 231 patients from GRI, 79 (34%) underwent NACRT for LARC. Most were male (58%), aged over 65 (51%) with mid- to upper rectal tumours (56%) and clinical T3/4 (95%), node-positive (77%) disease. pCR occurred in 10 patients (13%). Trends were noted between higher clinical T stage and poor response by Royal College of Pathologist’s TRG (*p* = 0.021) and tumour height > 5 cm and poor response by Mandard TRG (0.068). In the SMH cohort, 49 of 333 (15%) patients underwent NACRT; 8 (16%) developed a pCR. AC was not associated with NACRT response in either cohort.

**Conclusions:**

AC was not associated with NACRT response in this cohort. Larger contemporary cohorts are required to better assess host determinants of NACRT response and develop predictive models to improve patient selection.

**Supplementary Information:**

The online version contains supplementary material available at 10.1007/s00432-021-03570-1.

## Introduction

The management of rectal cancer has evolved significantly. The introduction of total mesorectal excision (TME) in the 1980s contributed to a substantial decline in local recurrence rates (Heald and Ryall 1986). Further improvements in local control have been gained through the use of radiotherapy in combination with a radio-sensitising agent prior to surgery in those with locally advanced (T stage 3 or 4 and/or node-positive) disease (Kapiteijn et al. 2001; Sauer et al. 2012). In 15–20% of patients, a pathologic complete response (pCR) occurs where no viable tumour is found on histological examination of the resection specimen (Maas et al. 2010). On this basis, the concept of non-operative management was described (Habr-Gama et al. 2004). Close observation of patients with evidence of a clinical complete response (cCR) on imaging and endoscopy following neoadjuvant chemoradiotherapy (NACRT) is now used in selected cases, avoiding the morbidity of surgery while providing comparable oncologic outcomes (Maas et al. 2011; Renehan et al. 2016; Dattani et al. 2018). However, TME following NACRT remains the standard of care for patients with locally advanced rectal cancer (LARC) who have an incomplete response or who opt for operative management (Gollins et al. 2017).

Grading of the response to NACRT has become a critical component of the management of LARC. The exponential rise in the use of magnetic resonance imaging (MRI) for locoregional staging pre- and post-NACRT has led to the development of tumour regression grades (TRGs) based on MRI findings (Battersby et al. 2016). The reference standard remains the degree of tumour regression present within the histological resection specimen which can be graded by a variety of pathological TRGs (Mandard et al. 1994; Rödel et al. 2005; Ryan et al. 2005). Observational studies attempting to correlate pathological and MRI-derived TRGs have, however, shown low levels of agreement (Chetty et al. 2012; Sclafani et al. 2017). Attempts to define the genotype associated with cCR in patients undergoing NACRT have been made, but factors such as intra-tumoural heterogeneity as well as the resource implications of gene sequencing techniques have prevented clinical translation (Lopes-Ramos et al. 2015). A simple, clinically relevant method of stratifying patients according to likely response to NACRT therefore represents a valuable tool in the management of patients with LARC.

It was recognised more than 60 years ago that hypoxia within the tumour microenvironment was associated with poorer response to radiotherapy (Thomlinson and Gray 1955). Hypoxia results from an imbalance between oxygen supply and demand during carcinogenesis and leads to the formation of abnormal tumour vasculature (Epstein et al. 2014). Systemic factors such as anaemia are also implicated in impaired oxygen delivery and reduced radiotherapy efficacy. Several studies have confirmed anaemia to be a negative prognostic indicator in response to NACRT for rectal cancer with greater clinical downstaging (Berardi et al. 2006) and higher rates of pathological regression (Box et al. 2005; Lee et al. 2009, 2012) reported in non-anaemic patients.

Vascular calcification of the aorta and iliac arteries has been associated with inferior outcomes following colorectal surgery including anastomotic leak and presumed to relate to impaired blood flow (Komen et al. 2011, Boersema et al. 2016, Eveno et al. 2016, Norooz et al. 2016, Pochhammer et al. 2018, Shen et al. 2019). As a marker of cardiovascular disease, aortic calcification may influence the dynamics of mesenteric flow by decreasing vessel pliability and reducing arterial diameter. Such macrovascular flow disturbance may compound the effects of systemic factors including anaemia and local factors including tumour hypoxia. It is possible that in patients with rectal cancer, significant aortic calcification (AC) may influence response to NACRT by limiting the flow of oxygenated blood to the tumour region. This study aimed to explore the relationship between host factors including the degree of AC present on pre-treatment imaging and response to NACRT in patients with LARC.

## Methods

Consecutive patients from Glasgow Royal Infirmary (GRI) with histologically proven rectal cancer who underwent neoadjuvant chemoradiation for LARC between 2008 and 2016 were identified from a prospectively maintained database. Exclusion criteria included patients who received short-course radiotherapy (SCRT) or systemic chemotherapy only. SCRT is used infrequently at our institution and is usually reserved for those with significant comorbidity who are deemed unfit for long-course format. To avoid potential selection bias and acknowledging that a very small number of patients received SCRT during the study period, these patients were excluded. LARC was defined as an involved circumferential margin (tumour, lymph node, lymphovascular and/or perineural disease ≤ 1 mm from the mesorectal fascia) (Loughrey et al. 2013). Referral for consideration of NACRT was made following formal discussion in the colorectal cancer multidisciplinary team (MDT) meeting.

All patients underwent staging following histopathologic confirmation of rectal adenocarcinoma using contrast-enhanced CT imaging of the thorax, abdomen and pelvis to rule out distant metastatic disease. In patients with no contraindication, locoregional staging of rectal cancer with pelvic MRI was performed. Tumour height was recorded as the distance in centimetres between the tumour and the anal verge on radiological staging and classified as low (< 5 cm), mid (5-10 cm) and upper (> 10 cm). Clinical stage was evaluated using digital rectal examination, endoscopic and radiological findings prior to treatment. Patients were re-staged and their imaging reviewed by the MDT on completion of NACRT. Rectal resection incorporating TME was performed using an open or laparoscopic approach approximately 8 weeks following completion of NACRT.

Clinico-pathological characteristics including details of the chemoradiation regimen, duration and dose were extracted from electronic patient records. Patients received long-course radiation at a dose of 45 Gy in 25 fractions over 5 weeks as standard. This was combined with a radio-sensitising agent, most commonly oral capecitabine. Patients with a history of significant cardiovascular disease were administered bolus 5-fluorouracil in weeks 1 and 5 of treatment in place of capecitabine.

To assess the degree of AC, a novel semi-quantitative visual assessment method was used. The derivation of this method and rationale for its use have previously been described (Knight et al. 2020). Briefly, calcification in two aortic territories was evaluated: the proximal aorta at the level of the SMA (proximal AC) and the distal aorta at the level of the aortoiliac bifurcation (distal AC). Both regions were considered separately, and a score of 0 to 4 assigned according to the number of calcified quadrants visible. A maximum score of 4 was possible for proximal AC. For distal AC, a score of 0 to 4 was possible for each of the three vessels including the distal aorta immediately proximal to the bifurcation and each common iliac artery at their origin. These were summed to provide a combined distal AC score with a maximum of 12 possible. Similar to previously published work, the degree of calcification was grouped into categories using the median:  absent (score 0), minor (less than median) or major (greater than median) (Harbaugh et al. 2013). A sample of 30 scans was analysed by five observers to assess inter-rater reliability in the GRI cohort, and by two observers in the St Mark’s cohort.

Pathological data were derived from reports issued at the time of resection. Tumours were staged using the Tumour, Nodes, Metastases (TNM) classification (Sobin and Fleming 1997) and according to the Royal College of Pathologists Dataset 2014 (Loughrey et al. 2013). Response to NACRT was determined using T- and N-downstaging, the degree of histopathologic tumour regression and the Neoadjuvant Rectal (NAR) score (George et al. 2015).

The reporting pathologist’s impression of response to preoperative therapy was recorded retrospectively using the tumour regression score advocated by the AJCC (Ryan et al. 2005). In addition, the degree of tumour regression was assessed retrospectively using the Mandard (Mandard et al. 1994) and Rödel (Rödel et al. 2005) grading systems. The Mandard tumour regression grade (TRG) uses a semi-quantitative approach to classify the proportion of residual cancer to scar tissue in the resection specimen. Similarly, the Rödel TRG assesses the amount of viable tumour in relation to the amount of fibrosis. The features of each TRG are outlined in Table 1.

**Table 1:** Tumour regression grades and the corresponding histopathological criteria

Tumour regression grade	Score	Description
Royal college of pathologists	0	No viable cancer cells (complete response)
	1	Single cells or rare small groups of cancer cells (near-complete response)
	2	Residual cancer with evident tumour regression, but more than single cells or rare small groups of cancer cells (partial response)
	3	Extensive residual cancer with no evident tumour regression (poor or no response)
Mandard	1	Complete regression—absence of residual cancer and fibrosis
	2	Presence of rare residual cancer
	3	An increase in the number of residual cancer cells, but predominantly fibrosis
	4	Residual cancer outgrowing fibrosis
	5	Absence of regressive changes
Rödel	Poor (0–1)	No regression or dominant tumour mass with obvious fibrosis and/or vasculopathy
	Intermediate (2–3)	Dominant fibrotic change with few tumour cells or groups (easy to find) or very few tumour cells in fibrotic tissue with or without mucous substance
	Complete (4)	No tumour cells, only fibrotic mass (total regression or response)

The Neoadjuvant Rectal (NAR) score, a surrogate endpoint developed for use in clinical trials to predict long-term outcome following NACRT for rectal cancer (George et al. 2015), was calculated for each patient using pre-treatment data. The formula incorporates clinical T stage and pathologic T and N-stage to produce a score between 0 and 100. Lower scores are suggested to indicate short-term benefit which may relate to improved survival. The difference between the pre-treatment clinical and post-treatment pathologic T- and N-stage were used to assess T- and N-downstaging.

A cohort of 333 LARC patients at St Mark’s Hospital and Academic Institute was identified from a prospectively maintained database between May 2007 and November 2016. Of them, 49 patients underwent NARCT following discussion of their cases at the local colorectal cancer MDT. Upon completion of the NACRT they underwent a TME for LARC. Data on clinical and radiological staging were not available; therefore, pCR rates were used to assess NACRT response. AC was assessed by one rater (ID). A sample of 30 scans was scored separately by two raters (KK, ID) to assess inter-rater reliability.

### Statistical analysis

Baseline characteristics were grouped according to standard thresholds and summarised using descriptive statistics. The intraclass correlation coefficient (ICC) was used to compare inter-rater reliability. ICC estimates and their 95% confident intervals were calculated based on a mean-rating, average measures, 2-way mixed-effects model. An ICC less than 0.5 was considered poor, between 0.5 and 0.75 moderate, 0.75–0.9 good and greater than 0.9 excellent. Associations between clinico-pathological characteristics and response to NACRT were assessed using Chi-squared test for association, Mantel–Haenszel or Fisher’s exact test where appropriate. Statistical analysis was performed using SPSS software (version 26, IBM, Armonk, NY). Ethical approval for the study was provided by the West of Scotland Research Ethics Committee (17-WS-0200). The need for patient consent was waived due to the retrospective nature of the study.

## Results

**Fig. 1:**
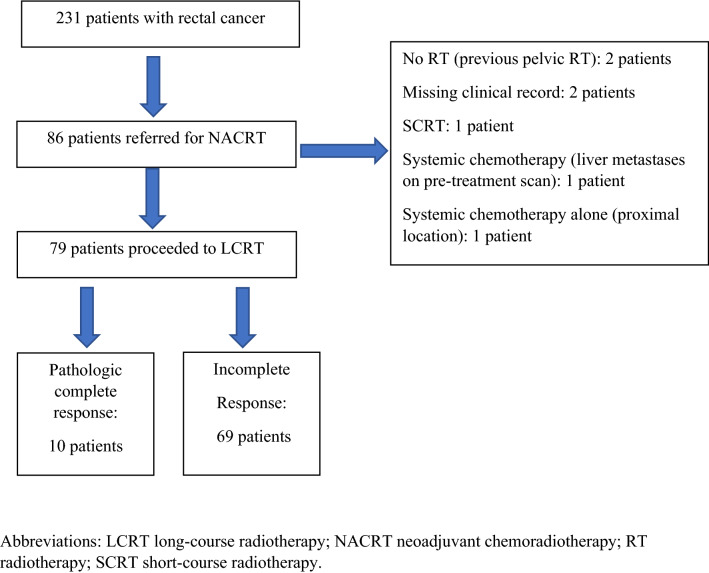
Flow diagram of patients from GRI undergoing treatment for rectal cancer between 2008 and 2016

Between 2008 and 2016, 231 patients underwent rectal cancer resection with curative intent at Glasgow Royal Infirmary Fig. 1. Of these, 86 patients were considered to have LARC on baseline clinical, imaging and endoscopic evaluation and were referred for consideration of NACRT following MDT discussion. In total, 79 patients proceeded to NACRT. Exclusions included two patients who had previously undergone pelvic radiotherapy for testicular and prostate cancer respectively, two patients with missing clinical records, two patients who received neoadjuvant systemic chemotherapy alone and one patient who underwent short-course radiotherapy.

The baseline characteristics of patients in the GRI cohort are displayed in Table 2. The majority of patients were male (*n* = 46, 58%), aged over 65 years (*n* = 40, 51%) and had a history of smoking (*n* = 44, 56%). Most patients had cT stage 3 or greater tumours (*n* = 75, 95%) in the mid- and upper rectum (*n* = 44, 56%) and node-positive disease (*n* = 60, 76%).

**Table 2:** Baseline demographics of patients undergoing neoadjuvant chemoradiation (*n* = 79)

Variable		*n* (%)
Age	< 65	39 (49)
	65–75	33 (42)
	> 75	7 (9)
Gender	Female	33 (42)
	Male	46 (58)
ASA grade	1	20 (25)
	2	38 (48)
	3	20 (25)
	4	1 (1)
BMI^a^	< 30	65 (82)
	> 30	13 (17)
Smoking history	No	35 (44)
	Yes	44 (56)
Tumour height (distance from anal verge, cm)	< 5	35 (44)
	5–10	26 (33)
	> 10	18 (23)
cT stage	2	4 (5)
	3	61 (77)
	4	14 (18)
cN stage^a^	0	18 (23)
	1	23 (29)
	2	37 (47)

*ASA* American Society of Anaesthesiology, *BMI* body mass index, *cT/N* clinical tumour/node stage, *NACRT* neoadjuvant chemoradiotherapy

^a^Missing cases 1

The NACRT regimen consisted of long-course radiotherapy (25 fractions of 45 Gy delivered over 5 weeks) in combination with oral capecitabine in 66 patients (84%). In 13 patients (16%), 5-fluorouracil (5-FU) was administered during weeks 1–5 of radiotherapy; 3 patients received concurrent folinic acid. NACRT was associated with toxicities in 23 patients (29%), with dose reductions or treatment interruptions occurring in 8 patients (10%).

MRI following NACRT was carried out in 21 patients (27%). All patients proceeded to surgery following NACRT. Surgical resection was performed by abdominoperineal resection in 40 patients (51%), anterior resection in 35 patients (44%) and Hartmann’s procedure in 4 patients (5%). All but one patient who underwent anterior resection had a primary anastomosis.

A pCR was reported in 10 patients (13%). The majority of patients who had a pCR had low rectal tumours (60%). In those with an incomplete response, T-downstaging occurred in 26 patients (38%) and N-downstaging in 34 patients (49%). Response to NACRT graded by the RCP TRG was complete in 10 patients (13%), near complete in 16 patients (20%), partial in 31 patients (39%) and poor in 22 (28%). Response to NACRT graded by the Mandard TRG was reported as complete in 11 patients (14%), rare residual cancer in 14 patients (18%), predominantly fibrosis in 13 patients (17%), residual cancer outgrowing fibrosis in 21 patients (26%) and absence of regression in 20 patients (25%). Response to NACRT graded by the Rödel TRG was complete in 10 patients (13%), intermediate in 40 (51%) and poor in 29 (37%). The NAR score was less than 8 in 12 patients (15%), 8 to 16 in 43 patients (54%) and greater than 16 in 24 patients (30%).

Associations between baseline characteristics including age, gender, pre-treatment haemoglobin level, cT stage, cN stage, tumour height and response to NACRT are displayed in Tables 3, 4, 5 and 6. No associations between pre-treatment host or tumour characteristics and pCR were evident. A statistically significant association was noted between lower cT stage tumours and complete or intermediate response to NACRT using the RCP TRG (*p* = 0.021). A non-significant trend between tumour height < 5 cm and complete response as graded by the Mandard TRG was noted. Expected associations between higher cT stage and degree of T-downstaging and nodal positivity and degree of N-downstaging were noted. No further statistically significant associations between baseline characteristics and response to NACRT using the Mandard TRG, Rödel TRG or T-downstaging were evident. A higher NAR score was associated with higher cN stage (*p* = 0.002) and tumour height < 5 cm (*p* = 0.002).

**Table 3:** Associations between baseline clinico-pathological characteristics and response to NACRT by histopathological response

		Incomplete response *n* = 69	Complete response *n* = 10	*p* value
Age (years)	< 65	36 (92)	3 (8)	0.608
	65–75	26 (79)	7 (21)	
	> 75	7 (100)	0 (0)	
Gender	Female	29 (88)	4 (12)	0.592
	Male	40 (87)	6 (13)	
Pre-NACRT haemoglobin (g/L)^a^	Normal	59 (86)	10 (14)	0.236
	Low	10 (100)	0 (0)	
cT stage	2–3	55 (84)	10 (16)	0.124
	4	14 (100)	0 (0)	
cN stage^b^	0	14 (78)	4 (22)	0.167
	1–2	54 (90)	6 (10)	
Tumour height (cm)	< 5	29 (83)	6 (17)	0.226
	5–10	23 (88)	3 (12)	
	> 10	17 (94)	1 (6)	

*cT/N* clinical tumour/node stage, *NACRT* neoadjuvant chemoradiotherapy

^a^Normal range 130–180 g/L for males, 110–165 g/L for females

^b^Nodal stage data missing for 1 patient

**Table 4:** Associations between baseline clinico-pathological characteristics and response to NACRT graded by the Royal College of Pathologists, Mandard and Rödel tumour regression grades (TRG)

		Royal College of Pathologists TRG			*p* value	Mandard TRG			*p* value	Rödel TRG			*p* value
		0 *n* = 10	1–2 *n* = 47	3 *n* = 22		1 *n* = 11	2–4 *n* = 48	5 *n* = 20		4 *n* = 10	2–3 *n* = 40	1 *n* = 29	
Age (years)	< 65	3 (8)	26 (67)	10 (25)	0.969	4 (10)	25 (64)	10 (26)	0.704	3 (8)	22 (56)	14 (36)	0.733
	65–75	7 (21)	16 (49)	10 (30)		7 (21)	17 (51)	9 (27)		7 (21)	13 (39)	13 (39)	
	> 75	0 (0)	5 (71)	2 (29)		0 (0)	6 (86)	1 (14)		0 (0)	5 (71)	2 (28)	
Gender	Female	4 (12)	17 (52)	12 (36)	0.273	4 (12)	18 (55)	11 (33)	0.233	4 (12)	14 (42)	15 (46)	0.293
	Male	6 (13)	30 (65)	10 (22)		7 (15)	30 (65)	9 (20)		6 (13)	26 (57)	14 (30)	
Pre-NACRT haemoglobin^a^	Normal	10 (14)	39 (56)	20 (29)	0.794	11 (16)	40 (58)	18 (26)	0.638	10 (15)	33 (48)	26 (38)	0.762
	Low	0 (0)	8 (80)	2 (20)		0 (0)	8 (80)	2 (20)		0 (0)	7 (70)	3 (30)	
cT stage	2–3	10 (15)	40 (62)	15 (23)	0.021	10 (15)	40 (62)	15 (23)	0.253	10 (15)	33 (51)	22 (34)	0.107
	4	0 (0)	7 (50)	7 (50)		1 (7)	8 (57)	5 (36)		0 (0)	7 (50)	7 (50)	
cN stage^b^	0	4 (22)	10 (56)	4 (22)	0.270	4 (22)	12 (67)	2 (11)	0.079	4 (22)	9 (50)	5 (28)	0.201
	1–2	6 (10)	37 (62)	17 (28)		7 (12)	35 (58)	18 (30)		6 (10)	31 (52)	23 (38)	
Tumour height (cm)	< 5	6 (17)	20 (57)	9 (26)	0.294	7 (20)	22 (63)	6 (17)	0.068	6 (17)	18 (52)	11 (31)	0.276
	5–10	3 (11)	16 (62)	7 (27)		3 (11)	15 (58)	8 (31)		3 (12)	12 (46)	11 (42)	
	> 10	1 (6)	11 (61)	6 (33)		1 (6)	11 (62)	6 (33)		1 (6)	10 (56)	7 (39)	

*cT/N* clinical tumour/node stage, *NACRT* neoadjuvant chemoradiotherapy

^a^Normal range 130–180 g/L for males, 110–165 g/L for females

^b^Nodal stage data missing for 1 patient

**Table 5:** Associations between baseline clinico-pathological characteristics and response to NACRT by T- and N-downstaging

		T-downstaging		*p* value	N-downstaging		*p* value
		No *n* = 43	Yes *n* = 36		No *n* = 39	Yes *n* = 39	
Age (years)	< 65	22 (56)	17 (44)	0.623	21 (54)	18 (46)	0.569
	65–75	15 (46)	18 (54)		13 (41)	19 (59)	
	> 75	6 (86)	1 (14)		5 (71)	2 (28)	
Gender	Female	20 (61)	13 (39)	0.351	20 (61)	13 (39)	0.109
	Male	23 (50)	23 (50)		19 (42)	26 (58)	
Pre-NACRT haemoglobin^a^	Normal	39 (56)	30 (44)	0.327	33 (49)	35 (51)	0.598
	Low	4 (40)	6 (60)		5 (50)	5 (50)	
cT stage	2–3	39 (60)	26 (40)	0.032	29 (45)	36 (55)	0.134
	4	4 (29)	10 (71)		9 (69)	4 (31)	
cN stage^b^	0	8 (44)	10 (56)	0.299	18 (100)	0 (0)	< 0.001
	1–2	35 (58)	25 (42)		21 (35)	39 (65)	
Tumour height (cm)	< 5	15 (43)	20 (57)	0.136	20 (59)	14 (41)	0.395
	5–10	17 (65)	9 (35)		9 (35)	17 (65)	
	> 10	11 (61)	7 (39)		9 (50)	9 (50)	

*cT/N* clinical tumour/node stage, *NACRT* neoadjuvant chemoradiotherapy

^a^Normal range 130–180 g/L for males, 110–165 g/L for females

^b^Nodal stage data missing for 1 patient

**Table 6:** Associations between baseline clinico-pathological characteristics and response to NACRT by Neoadjuvant Rectal (NAR) score

		NAR score			*p* value
		< 8 *n* = 12	8–16 *n* = 43	> 16 *n* = 24	
Age (years)	< 65	3 (8)	25 (25)	11 (28)	0.765
	65–75	8 (24)	15 (46)	10 (30)	
	> 75	1 (14)	3 (43)	3 (43)	
Gender	Female	5 (15)	15 (46)	13 (39)	0.303
	Male	7 (15)	28 (61)	11 (24)	
Pre-NACRT haemoglobin (g/L)^a^	Normal	12 (17)	35 (51)	22 (32)	0.806
	Low	0 (0)	8 (80)	2 (20)	
cT stage	2–3	11 (17)	35 (54)	19 (29)	0.404
	4	1 (7)	8 (57)	5 (36)	
cN stage^b^	0	6 (33)	11 (61)	1 (6)	0.002
	1–2	2 (10)	31 (52)	23 (38)	
Tumour height (cm)	< 5	8 (23)	23 (66)	4 (11)	0.002
	5–10	3 (12)	12 (46)	11 (42)	
	> 10	1 (6)	8 (44)	9 (50)	

*cT/N* clinical tumour/node stage, *NACRT* neoadjuvant chemoradiotherapy

^a^Normal range 130–180 g/L for males, 110–165 g/L for females

^b^Nodal stage data missing for 1 patient

The associations between the degree of AC and response to NACRT are shown in Table 7. Proximal AC was absent in 45 patients (57%), minor in 19 patients (24%) and major in 15 patients (19%) while distal AC was absent in 25 patients (32%), minor in 23 patients (29%) and major in 31 patients (39%). There were no statistically significant associations between the degree of proximal or distal AC and response to NACRT as measured by pCR rates, RCP, Mandard and Rödel TRGs, T-downstaging, N-downstaging or NAR score. The ICC for inter-rater reliability for proximal AC was 0.89 (95% CI 0.82–0.94) and for distal AC 0.92 (95% CI 0.87–0.96).

**Table 7:** Comparison of response to NACRT by the degree of calcification

		Proximal AC				Distal AC			
		None *n* = 45	Minor *n* = 19	Major *n* = 15	*p* value	None *n* = 25	Minor *n* = 23	Major *n* = 31	*p* value
pCR	No	39 (57)	17 (25)	13 (18)	0.931	23 (33)	18 (26)	28 (41)	0.923
	Yes	6 (60)	2 (20)	2 (20)		2 (20)	5 (50)	3 (30)	
TRG—Royal College of Pathologists	Complete (0)	6 (60)	2 (20)	2 (20)	0.720	2 (20)	5 (50)	3 (30)	0.652
	Intermediate (1–2)	28 (60)	10 (21)	9 (19)		17 (36)	12 (26)	18 (38)	
	Poor (3)	11 (50)	7 (32)	4 (18)		6 (27)	6 (27)	10 (46)	
TRG—Mandard	Complete (1)	7 (64)	2 (18)	2 (18)	0.923	3 (27)	5 (46)	3 (27)	0.473
	Intermediate (2–4)	27 (56)	11 (23)	10 (21)		16 (33)	14 (29)	18 (38)	
	Poor (5)	11 (55)	6 (30)	3 (15)		6 (30)	4 (20)	10 (50)	
TRG—Rödel	Complete (4)	6 (60)	2 (20)	2 (20)	0.793	2 (20)	5 (50)	3 (30)	0.606
	Intermediate (2–3)	24 (60)	8 (20)	8 (20)		15 (38)	10 (25)	15 (38)	
	Poor (0–1)	15 (52)	9 (31)	5 (17)		8 (28)	8 (28)	13 (45)	
T-downstaging	No	22 (51)	14 (33)	7 (16)	0.704	13 (30)	12 (28)	18 (42)	0.642
	Yes	23 (64)	5 (14)	8 (22)		12 (33)	11 (31)	13 (36)	
N-downstaging	No	22 (58)	10 (26)	6 (16)	0.592	10 (26)	13 (34)	15 (40)	0.580
	Yes	22 (55)	9 (23)	9 (23)		15 (38)	9 (22)	16 (40)	
NAR score	Low (< 8)	6 (50)	2 (17)	4 (33)	0.924	2 (17)	5 (42)	5 (42)	0.555
	Intermediate (8–16)	27 (63)	10 (23)	6 (14)		15 (35)	11 (26)	17 (40)	
	High (> 16)	12 (50)	7 (29)	5 (21)		8 (33)	7 (29)	9 (38)	

*AC* aortic calcification, *NAR* Neoadjuvant Rectal score, *pCR* pathologic complete response, *TRG* tumour regression grade

**Fig. 2:**
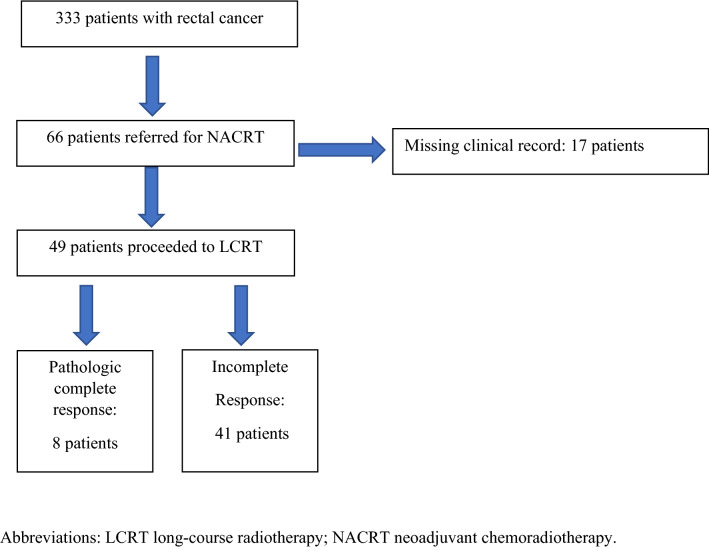
Flow diagram of patients from St Mark’s Hospital undergoing treatment for rectal cancer between 2008 and 2016

Between 2007 and 2016, 333 patients with available CT imaging underwent rectal cancer resection with curative intent at St Mark’s Hospital Fig. 2. Of these, 49 patients proceeded to NACRT. The baseline characteristics of patients in the study cohort are displayed in Supplementary Table 1a. The majority of patients were male (*n* = 37, 75%), aged less than 65 years (*n* = 29, 59%) and were ASA grade 1 or 2 (*n* = 43, 87%). A pCR occurred in 8 patients (16%).

Proximal AC was absent in 36 patients (74%), minor in 6 patients (12%) and major in 7 patients (14%) while distal AC was absent in 20 patients (41%), minor in 15 patients (31%) and major in 14 patients (28%). For inter-rater reliability, the ICC for proximal AC was 0.92 (95% CI 0.84–0.96) and for distal AC was 0.88 (95% CI 0.75–0.94).

There were no statistically significant associations between the development of a pCR and age, gender or the degree of proximal or distal AC in the St Mark’s cohort (Supplementary Table 1b). This remained the case when patients from both cohorts were pooled (Supplementary Table 1c).

## Discussion

In this study, neither the degree of proximal or distal aortic calcification was associated with response to NACRT in patients with margin-threatening rectal cancer. Distal AC was more common than proximal AC in both cohorts. When compared to patients with rectal cancer who did not undergo NACRT (data not shown), both proximal and distal calcification rates were similar, suggesting the degree of calcification in this study cohort was representative of that in patients with rectal cancer who proceeded directly to surgery.

The absence of an association between the degree of calcification and NACRT response may be related to several factors. The small number of patients with a complete response (*n* = 10, 13% GRI, *n* = 8, 16% SMH) is likely to limit our ability to detect a relationship. This may also underlie the lack of association between treatment response and anaemia, present in only 10 patients from the GRI cohort. Using the TRGs, most patients were categorised as having an intermediate response, making differentiation of factors predisposing to a complete or poor response difficult. However, binary response measures such as T- and N-downstaging were not associated with the degree of aortic calcification.

The optimal endpoint for assessment of response to neoadjuvant therapy is a source of ongoing debate. Tumour regression grades are commonly associated with high rates of interobserver variability (Chetty et al. 2012). The variable diagnostic performance of MRI (Horvat et al. 2018) and the need for multiple integrated sequences to improve predictive capacity for pCR (Maas et al. 2015; Hötker et al. 2016) limit their use. Moreover, wide variation in pCR rates across institutions has been attributed to differences in the thoroughness of pathological examination (Chow et al. 2019). The NAR score was developed as a surrogate endpoint for use in clinical trials which involve assessment of response to NACRT, but its predictive value has been disputed in subsequent studies (van der Valk et al. 2019). The use of multiple metrics of tumour response was therefore undertaken in this study. However, no association between these measures and the degree of aortic calcification was evident in either cohort. It was notable that variables such as tumour height which are associated with NACRT response in other series did not consistently show significant associations with measures of NACRT response, suggesting an expanded sample size is required to validate the study findings.

Relatively few studies have examined the relationship between aspects of comorbidity and treatment response. Anderson and colleagues found hypertension to be the sole component of the metabolic syndrome (hypertension, obesity, hypertriglyceridaemia, elevated fasting glucose and reduced HDL cholesterol) associated with reduced odds of complete response in a cohort of 102 patients with a pCR rate of 17% (Anderson et al. 2016). However, a limited number of patients had metabolic syndrome in this study (6%) while 50% had hypertension which was poorly defined in the study methods. Blood pressure was not measured before or during NACRT and it is possible that patients with a history of hypertension were normotensive on minimal medication, limiting the reliability of the findings. A further study examining the impact of diabetes mellitus on response to NACRT in 102 patients with rectal cancer reported similar rates of tumour downstaging between diabetic and non-diabetic patients but a difference in pCR rates (Caudle et al. 2008). None of the patients with diabetes were found to have a complete response compared with 24% of their non-diabetic counterparts. Although the small cohort size also limits generalisability, the possibility that microvascular rather than macrovascular calcification, as is common in diabetes, could influence radiotherapy response warrants further exploration.

Comparison of the tumour microenvironment characteristics and the degree of calcification in the patients in this study would have provided clarity on the relationship between aortic calcification and markers of tumour hypoxia. However, availability of tissue for analysis from patients within the cohort was limited. Future studies examining NACRT response in relation to patient and tumour microenvironment characteristics are required. Moreover, the paucity of data on the effect of comorbidity on NACRT response suggests integration of comorbidity indices would provide more context to assess the clinical relevance of aortic calcification in patients with rectal cancer.

Several practical aspects must also be considered: the use of NACRT for LARC within the UK is variable (Morris et al. 2016) as demonstrated by the differences in the proportion of patients undergoing NACRT in the two cohorts described here (34% GRI, 15% St Mark’s). In addition, use of NACRT in the UK is generally reserved for poor prognosis tumours, i.e. low margin-threatening node-positive tumours whereas NACRT in North America is not restricted to such so-called “ugly” tumours. Debate continues regarding the optimal format of NACRT (short-course radiotherapy versus long-course chemoradiation) while trials are underway examining the addition of systemic chemotherapy to the neoadjuvant treatment schedule (total neoadjuvant therapy) (Garcia-Aguilar et al. 2020). Such differences in treatment indication and format necessitate examination of the influence of host factors on NACRT response in a large contemporary cohort. Techniques such as propensity-score matching may be required to enable reliable comparison between cohorts from UK, Europe and North America.

As described, this study has several limitations. In addition, data on tumour volume pre- and post-NACRT as a response metric were limited. Similarly, only a small number of patients had both pre- and post-NACRT MR imaging available to enable MRI-based assessment of treatment response. The use of 5-FU in place of capecitabine in patients with established cardiovascular disease among the GRI cohort may also have influenced response to NACRT. The St Mark’s dataset contained a small proportion of patients undergoing NACRT and was limited by the absence of clinical staging and MRI data, restricting response to treatment analysis to pCR only. However, the reference standard for NACRT response remains histopathological examination, suggesting additional proxy measures may have little impact on the study findings.

In conclusion, in the absence of an available larger cohort in which to examine NACRT response in relation to host characteristics, these data suggest that aortic calcification does not appear to significantly influence treatment response. Further work to assess the degree of hypoxia within the tumour microenvironment may provide additional information on the relationship between vascular calcification, tumour hypoxia and NACRT response.

## Supplementary Information

Below is the link to the electronic supplementary material.

Supplementary file1 (DOCX 26 KB)

## Data Availability

The data that support the findings of this study are available on reasonable request from the corresponding author. The data are not publicly available due to privacy and ethical restrictions.
