# Supplementary material for: Vascular calcification and response to neoadjuvant therapy in locally advanced rectal cancer: an exploratory study
Source: J Cancer Res Clin Oncol. 2021 Mar 12;147(11):3409–20. doi: 10.1007/s00432-021-03570-1 (PMC8484095; doi:10.1007/s00432-021-03570-1)
Supplement: Supplementary file 1 — Supplementary file1 (DOCX 26 KB) [file 432_2021_3570_MOESM1_ESM.docx]

**Supplementary Table 1a:** Baseline demographics of patients undergoing neoadjuvant chemoradiation (St Mark’s cohort, n=49).

| **Variable** | | n (%) |
| --- | --- | --- |
| **Age (years)^a^** | **<65** | 29 (59) |
|  | **65 - 75** | 10 (20) |
|  | **>75** | 9 (18) |
| **Gender** | **Female** | 12 (25) |
|  | **Male** | 37 (75) |
| **ASA grade^a^** | **1** | 10 (20) |
|  | **2** | 33 (67) |
|  | **3** | 4 (8) |
|  | **4** | 1 (2) |
| **BMI^a^** | **<30** | 38 (78) |
|  | **>30** | 6 (12) |

Abbreviations: ASA American Society of Anaesthesiologists, BMI body mass index.

^a^ Missing cases: Age (n=1), ASA (n=1), BMI (n=5).

**Supplementary Table 1b:** Associations between baseline clinico-pathological characteristics and response to NACRT by histopathological response in St Mark’s cohort.

|  | | **Incomplete response**  **n = 41** | **Complete response**  **n = 8** | **p-value** |
| --- | --- | --- | --- | --- |
| **Age (years)^a^** | **< 65** | 22 (76) | 7 (24) | 0.235 |
|  | **65 – 75** | 10 (100) | 0 (0) |  |
|  | **> 75** | 8 (89) | 1 (11) |  |
| **Gender** | **Female** | 9 (75) | 3 (25) | 0.386 |
|  | **Male** | 32 (86) | 5 (14) |  |
| **Proximal AC** | **None** | 29 (81) | 7 (19) | 0.621 |
|  | **Minor** | 6 (100) | 0 (0) |  |
|  | **Major** | 6 (86) | 1 (14) |  |
| **Distal AC** | **None** | 15 (75) | 5 (25) | 0.175 |
|  | **Minor** | 13 (87) | 2 (13) |  |
|  | **Major** | 13 (93) | 1 (7) |  |

Abbreviations: AC – aortic calcification.

^a^ Missing cases: Age (n= 1)

**Supplementary Table 1c:** Associations between baseline clinico-pathological characteristics and response to NACRT by histopathological response in combined Glasgow Royal and St Mark’s cohort (n=128).

|  | | **Incomplete response**  **n = 110** | **Complete response**  **n = 18** | **p-value** |
| --- | --- | --- | --- | --- |
| **Age (years)^a^** | **< 65** | 58 (85) | 10 (24) | 0.557 |
|  | **65 – 75** | 36 (84) | 7 (16) |  |
|  | **> 75** | 15 (94) | 1 (6) |  |
| **Gender** | **Female** | 38 (84) | 7 (16) | 0.721 |
|  | **Male** | 72 (87) | 11 (13) |  |
| **Proximal AC** | **None** | 68 (84) | 13 (16) | 0.575 |
|  | **Minor** | 23 (92) | 2 (8) |  |
|  | **Major** | 19 (86) | 3 (14) |  |
| **Distal AC** | **None** | 38 (84) | 7 (16) | 0.365 |
|  | **Minor** | 31 (82) | 7 (18) |  |
|  | **Major** | 41 (91) | 4 (9) |  |

Abbreviations: AC – aortic calcification.

^a^ Missing cases: Age (n= 1)
